# Metformin overcomes resistance to cisplatin in triple-negative breast cancer (TNBC) cells by targeting RAD51

**DOI:** 10.1186/s13058-019-1204-2

**Published:** 2019-10-22

**Authors:** Jung Ok Lee, Min Ju Kang, Won Seok Byun, Shin Ae Kim, Il Hyeok Seo, Jeong Ah. Han, Ji Wook Moon, Ji Hae Kim, Su Jin Kim, Eun Jung Lee, Serk In Park, Sun Hwa Park, Hyeon Soo Kim

**Affiliations:** 1 Department of Anatomy, Korea University College of Medicine, 126-1, Anam-dong 5-ga, Seongbuk-gu, Seoul, Republic of Korea; 2 Department of Biochemistry and Molecular Biology, Korea University College of Medicine, Seoul, Republic of Korea; 3 Department of Medicine, Vanderbilt University School of Medicine, Nashville, TN USA

**Keywords:** Cisplatin resistance, Metformin, RAD51, TNBC, Combination therapy

## Abstract

**Background:**

Chemotherapy is a standard therapeutic regimen to treat triple-negative breast cancer (TNBC); however, chemotherapy alone does not result in significant improvement and often leads to drug resistance in patients. In contrast, combination therapy has proven to be an effective strategy for TNBC treatment. Whether metformin enhances the anticancer effects of cisplatin and prevents cisplatin resistance in TNBC cells has not been reported.

**Methods:**

Cell viability, wounding healing, and invasion assays were performed on Hs 578T and MDA-MB-231 human TNBC cell lines to demonstrate the anticancer effects of combined cisplatin and metformin treatment compared to treatment with cisplatin alone. Western blotting and immunofluorescence were used to determine the expression of RAD51 and gamma-H2AX. In an in vivo 4T1 murine breast cancer model, a synergistic anticancer effect of metformin and cisplatin was observed.

**Results:**

Cisplatin combined with metformin decreased cell viability and metastatic effect more than cisplatin alone. Metformin suppressed cisplatin-mediated RAD51 upregulation by decreasing RAD51 protein stability and increasing its ubiquitination. In contrast, cisplatin increased RAD51 expression in an ERK-dependent manner. In addition, metformin also increased cisplatin-induced phosphorylation of γ-H2AX. Overexpression of RAD51 blocked the metformin-induced inhibition of cell migration and invasion, while RAD51 knockdown enhanced cisplatin activity. Moreover, the combination of metformin and cisplatin exhibited a synergistic anticancer effect in an orthotopic murine model of 4T1 breast cancer in vivo.

**Conclusions:**

Metformin enhances anticancer effect of cisplatin by downregulating RAD51 expression, which represents a novel therapeutic target in TNBC management.

## Background

Triple-negative breast cancer (TNBC), which represents 10–20% of all breast cancers, is characterized by a lack of expression of the estrogen steroid receptor (ER), progesterone steroid receptor (PR), and tyrosine kinase human epidermal growth factor receptor 2 (HER2) [1]. Compared to other cancer subtypes, TNBC tumors are more frequently diagnosed as aggressive, invasive, grade III, and lymph node-positive [2]; however, no effective targeted therapy is currently available for the treatment of TNBC. Although approximately 50% of all patients with TNBC respond to conventional chemotherapies [3, 4], the effectiveness of these treatments is limited by the development of drug resistance [5, 6].

Cisplatin is widely used to treat solid tumors, including breast, testicular, and ovarian cancers [7]. Cisplatin exerts its anticancer effects by inducing DNA double-strand breaks (DSBs) [8, 9]. Despite a consistent initial response, cisplatin treatment results in the development of chemoresistance. For example, patients who initially respond to cisplatin therapy often develop resistance due to activation of the homologous recombination (HR) DNA repair mechanism [10, 11]. Multiple mechanisms underlying the development of resistance include altered cellular accumulation [12], increased drug inactivation [13], and DNA repair [14].

Homologous recombination is an error-free DNA repair mechanism for DSBs that is activated when cells are exposed to genotoxic stress [15, 16]. RAD51 is a strand transferase that polymerizes into a nucleoprotein filament on single-stranded DNA and promotes DNA strand exchange with the undamaged homologous chromatid [17]. Because RAD51 is an integral component of the cellular DNA damage response, its suppression sensitizes cancer cells to DNA-damaging drugs [18, 19]. In contrast, high levels of RAD51 have been linked to elevated rates of DNA recombination and enhanced resistance to DNA-damaging chemotherapies and/or ionizing radiation [20, 21]. In addition, RAD51 facilitates TNBC metastasis [22], indicating that RAD51 is a therapeutic target for TNBC treatment.

Metformin (1,1-dimethylbiguanide hydrochloride), the most commonly prescribed oral antidiabetic medication, may be of benefit to diabetic cancer patients [23]. Notably, the breast cancer risk has been shown to be lower in diabetic patients treated with metformin than in those treated with other antidiabetic medications [24].. Metformin was shown to inhibit the DNA damage repair pathway in pancreatic cancer [25], p53-deficient colorectal cancer [26], and non-small cell lung cancer (NSCLC) cells [27] by downregulating RAD51, indicating the anticancer effects of metformin. In addition, increased glucose concentrations reduced the efficacy of metformin [28], implying that high glucose levels may negatively influence the anticancer efficacy of metformin. In our study, we also found that metformin decreased RAD51 expression more efficiently in culture conditions containing a normal glucose concentration (5 mM) than in conditions with high glucose concentrations (25 mM). Moreover, metformin also enhanced the therapeutic effect of cisplatin in ovarian cancer [29], nasopharyngeal carcinoma cells [30], lung tumors [31], and oral squamous carcinoma cells [32]. These observations led us to hypothesize that metformin may sensitize TNBC cells to cisplatin by downregulating RAD51 under physiological glucose concentrations. In the present study, we explored the therapeutic role of metformin and demonstrate that, in combination with cisplatin, metformin is effective TNBC treatment outcomes.

## Methods

### Reagents

Antibodies against RAD51 and phospho-H2AX (Ser^139^) were purchased from Abcam (Cambridge, UK). Antibodies against ubiquitin were purchased from Cell Signaling Technology (Danvers, MA, USA), while antibodies against β-actin were from Sigma-Aldrich (St. Louis, MO, USA). Anti-ERK1/2 and anti-phospho-ERK1/2 (Thr^202^/Tyr^204^) antibodies were procured from Santa Cruz Biotechnology (Santa Cruz, CA, USA). Horseradish peroxidase (HRP)-conjugated goat anti-rabbit IgG and goat anti-mouse IgG secondary antibodies were obtained from Enzo Life Sciences (Farmingdale, NY, USA). Cisplatin, metformin, MG132 (carbobenzoxy-Leu-Leu-leucinal), cycloheximide (CHX), PD98059, and lactacystin were obtained from Sigma-Aldrich. Protein A agarose beads were acquired from GE Healthcare (Piscataway, NJ, USA).

### Cell culture

MDA-MB-231 and Hs 578T human breast cancer cells (ATCC, Rockville, MD, USA) were maintained in Dulbecco’s high glucose (25 mM glucose) modified Eagle’s medium (DMEM) supplemented with 10% fetal bovine serum (FBS) (Gibco, Carlsbad, CA, USA), 100 U/mL penicillin, and 100 μg/mL streptomycin. MCF10A cells were grown in DMEM/F-12 medium (Gibco) containing 5% horse serum (Gibco), 20 ng/mL EGF, 0.5 mg/mL hydrocortisone, 100 ng/mL cholera toxin, 10 μg/mL insulin, 100 U/mL penicillin, and 100 μg/mL streptomycin. All cells were cultured at 37 °C in a humidified incubator with 5% CO_2_.

### MTT assay

Cell viability was measured by MTT assay. MDA-MB-231 and Hs 578T cells were seeded into 96-well plates at a density of 1 × 10^3^ cells/mL. Growth medium was replaced with normal (5.5 mM) glucose medium 24 h prior to treatment. Subsequently, MTT (0.5 mg/mL) was added and the cells were incubated for 2 h at 37 °C. The cells were then lysed with DMSO, and the absorbance at 540 nm was measured using a microplate reader (Bio-Rad, Hercules, CA, USA).

### Western blot analysis

The medium was removed, and cells were washed with ice-cold phosphate-buffered saline (PBS). The cells were then lysed in 100 μL of lysis buffer (50 mM Tris-HCl [pH 7.4], 1% Triton X-100, 0.25% sodium deoxycholate, 150 mM EDTA, 1 mM sodium orthovanadate [Na_3_VO_4_], 1 mM NaF, 1 mM phenylmethylsulfonyl fluoride [PMSF]). Proteins were resolved on 10% SDS-PAGE gels and transferred to nitrocellulose membranes. The membranes were blocked in 5% dry milk (w/v) for 1 h and then washed three times in TBST (Tris-buffered saline with Triton X-100). The membranes were incubated overnight at 4 °C with primary antibodies and then probed with an HRP-conjugated secondary antibody for 1 h. Blots were visualized using the Amersham Biosciences ECL Detection System (Amersham plc, GE Healthcare, Chicago, IL, USA).

### siRNA transfection for RAD51 knockdown

MDA-MB-231 and Hs 578T human breast cancer cells were seeded in six-well plates and transfected at 60% confluence with RAD51-targeting siRNA duplexes or a negative control siRNA (L-003530-00-0005; UAUCAUCGCCCAUGCAUCA, CUAAUCAGGUGGUAGCUCA, GCAGUGAUGUCCUGGAUAA, and CCAACGAUGUGAAGAAAUU) purchased from Dharmacon (Lafayette, CO, USA). For transfection, 5 μL of siRNA targeting human RAD51 (CR536559) and 5 μL of Lipofectamine were each diluted in 95 μL of reduced serum medium (Opti-MEM, Invitrogen, Carlsbad, CA, USA). The mixtures were incubated for 15 min before being added dropwise to the culture wells containing 800 μL of Opti-MEM to achieve a final siRNA concentration of 50 nM.

### Construction of pFLAG-RAD51

Human RAD51 was cloned into the BamHI and SalI sites of the pCMV-Tag 2C vector (Stratagene, San Diego, CA, USA). The cDNA from MDA-MB-231 cells was amplified by polymerase chain reaction (forward primer: 5′-CGGGATCCATGGCAATGCAGATGCAGC-3′; reverse primer: 5′-ACGGCGTCGACTCAGTCTTTGGCATCTCCCAC-3′), digested with BamHI and SacI, and then ligated to a linearized pCMV-Tag 2C vector. The construct was verified by DNA sequencing.

### Wound healing assay

Confluent cells were serum-starved for 12 h, after which a standardized cell-free area was introduced by scraping the monolayer with a sterile tip. Cells were imaged using a phase-contrast microscope. After intensive washing, fresh medium supplemented with 10% FBS containing both metformin and cisplatin was added. After incubation for 36 h, three random areas of cells were imaged. Migrated cells were quantified by manual counting, and the inhibition ratios were expressed as percentages of control cells.

### Invasion assay

The upper chamber of a Transwell insert (8-μm pore size) was coated with 100 μL of Matrigel (BD Biosciences, Bedford, MA, USA) and PBS, followed by drying for 30 min at 37 °C. Cells were suspended in serum-free medium (100 μL; 4 × 10^5^ cells/mL) and layered in the upper compartment of the chamber. The bottom chambers were supplemented with 500 μL of complete medium (10% FBS) containing the indicated concentrations of both metformin and cisplatin. After incubation for 24 h, the invading cells on the lower face were fixed in 4% paraformaldehyde and stained with crystal violet (Sigma-Aldrich). Random fields were counted, and representative images were obtained using an AxioCam HRC CCD camera (Carl Zeiss, Oberkochen, Germany).

### Immunoprecipitation

Cellular protein (1 mg) was mixed with 1 μg of anti-RAD51 rabbit monoclonal antibodies and incubated at 4 °C for 24 h. Immune complexes were captured with protein A sepharose (Amersham, Uppsala, Sweden) for an additional 3 h. The precipitated immune complexes were washed three times with wash buffer, resuspended in SDS sample buffer (125 mM Tris-HCl [pH 6.8], 20% [v/v] glycerol, 4% [w/v] SDS, 100 mM dithiothreitol, and 0.1% [w/v] bromophenol blue), and heated at 95 °C for 5 min prior to electrophoresis.

### Immunofluorescence staining

Cells were seeded in 12-well plates at a density of 5 × 10^4^ cells/well on a sterile coverslip. After treatment with metformin or cisplatin, the cells were washed with PBS, fixed in 4% formaldehyde, and permeabilized with 0.2% Triton X-100 in PBS for 15 min. After blocking with 2% bovine serum albumin (BSA) in PBS for 1 h at room temperature, the cells were incubated overnight with primary antibodies against RAD51 and γ-H2AX in blocking buffer at 4 °C. The cells were then washed in PBS and incubated with Alexa Fluor 488-conjugated chicken anti-rabbit IgG secondary antibodies (1:500, Invitrogen) and Alexa Fluor 568-conjugated donkey anti-rabbit antibodies (1:500, Invitrogen) for 1 h at room temperature. The cells were counterstained with Hoechst 33342 for 10 min before the final wash. Images were captured using a confocal microscope (Zeiss LSM 700 Meta, Carl Zeiss) at × 10 magnification.

### Experimental animals and tumor inoculation

Forty female BALB/c mice were randomly divided into four groups of 10 mice each. The mice in the control group were inoculated with 4T1 cells, while those in the metformin group were injected intraperitoneally with metformin (150 mg/kg body weight per day) for 21 days. The experiment was approved by the Korea University Institutional Animal Care and Use Committee (IACUC) and was performed according to the guidelines and regulations. The mice in the combination therapy group were injected intraperitoneally with metformin (150 mg/kg body weight per day) and cisplatin (3 mg/kg body weight once every 3 days) for 21 days. Mice in the cisplatin group were injected intraperitoneally with cisplatin (3 mg/kg body weight once every 3 days) starting from day 5 of tumor inoculation. The body weight of each mouse was determined daily during the entire experimental period. The 4T1 tumor cell suspension was diluted in PBS and injected subcutaneously (0.2 mL, 4 × 10^5^ cells/mouse) and bilaterally into the fourth pair of mammary fat pads of each mouse. All injections were administered in a 0.15-mL volume. Tumor growth was determined by measuring the tumor diameter in two dimensions with a caliper every 3 days, and the tumor volumes ([width^2^ × length]/2) were calculated. Body weight was recorded to monitor the side effects of the drugs. Breast tumors and gonadal fat pads were either homogenized to prepare tissue lysates for western blot analysis, or formalin-fixed, embedded in paraffin, and cut into 5-μM sections for immunohistochemistry (IHC).

### Immunohistochemical analysis

Paraformaldehyde (4%)-fixed samples were gradually dehydrated in a graded ethanol series and cleared in xylene using a Leica AS300S tissue processor (Leica Microsystems GmbH, Wetzlar, Germany). The samples were then infiltrated with paraffin and cut into 5-μm sections using a Leica RM2255 rotary microtome (Leica Microsystems GmbH). Representative blocks of paraffin-embedded tissues were dewaxed and rehydrated. Briefly, sections were deparaffinized, rehydrated, and washed in PBS. To block nonspecific binding, sections were incubated in 4% BSA-dextran for 1 h at 4 °C. Sections were incubated with anti-RAD51 antibodies diluted 1:200 in 1% BSA and 0.1% Nonidet P-40 in PBS overnight at 4 °C. The Vectastain ABC kit (Vector Labs, Burlingame, CA, USA) was used to amplify the signal using the avidin-biotin complex (ABC) method according to the manufacturer’s instructions. Peroxidase activity was visualized with 3,3′-diaminobenzidine (DAB; Darko, Carpinteria, CA, USA). Sections were lightly counterstained with hematoxylin, dehydrated through an ethanol series to xylene, and mounted. Slides were visualized and imaged using a light microscope equipped with a computer-controlled digital camera.

### Statistical analysis

Data are expressed as means ± SEM. One-way analysis of variance (ANOVA) was performed to compare multiple groups followed by Bonferroni’s post hoc test. A *P* value of 0.05 or lower was considered significant in all experiments. All analyses were performed using Sigma plot software (Systat Software Inc., San Jose, CA, USA). *P* values less than 0.05 were considered significant and were presented as #, ## vs. no treatment; ^#^*P* < 0.05, ^##^*P* < 0.01, ^###^*P* < 0.001, *P < 0.05, **P < 0.01, **P < 0.001 by one-way ANOVA followed by Bonferroni’s post hoc test.

## Results

### Metformin enhances cisplatin-mediated inhibitory effects on cell proliferation, migration, and invasion

**Fig. 1:**
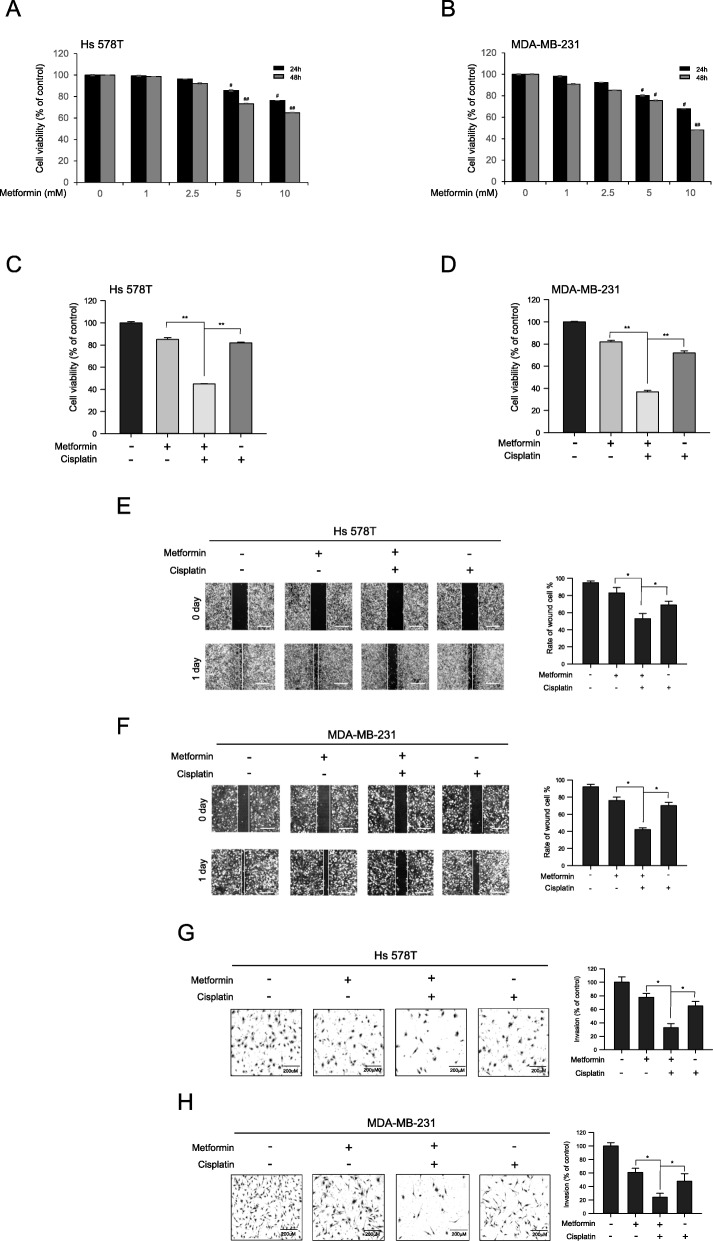
Metformin enhances the anticancer effects of cisplatin in TNBC cells. **a**, **b** Hs 578T and MDA-MB-231 cells were treated with metformin (1~10 mM) for 24 or 48 h in DMEM supplemented with 10% FBS and 5 mM glucose (i.e., normoglycemic conditions), followed by MTT assay. **c**, **d** Hs 578T and MDA-MB-231 cells were treated for 24 h with 5 mM metformin, 5 μM cisplatin, or a combination of metformin + cisplatin, followed by MTT assay. Cell viability was expressed as the percentage of viable cells in treated wells relative to the percentage of viable cells in control wells (100% viability). **e**, **f** Cultures of Hs 578T and MDA-MB-231 cells were wounded by scratching with a pipette tip and incubated with metformin (5 mM), cisplatin (5 μM), or a combination of 5 mM metformin + 5 μM cisplatin. Representative images of wound healing were obtained at the time of the scratch and after 24 h. **g**, **h** Invasiveness of Hs 578T and MDA-MB-231 cells was measured using a Matrigel Transwell assay following treatment with metformin (5 mM), cisplatin (5 μM), or a combination of 5 mM metformin + 5 μM cisplatin for 24 h. Cell invasion was quantified by staining and counting membrane-associated cells in the lower surface of the Transwell. Results represent the mean ± SEM of five independent experiments. #, ## vs. no treatment; ^#^*P* < 0.05, ^##^*P* < 0.01, ^###^*P* < 0.001, *P < 0.05, **P < 0.01, by one-way ANOVA followed by Bonferroni’s post hoc test

Metformin has previously been reported to significantly inhibit the growth of different cancer cells cultured in normoglycemic conditions, i.e., at glucose concentrations between 4 and 6 mM [28]. We assessed the effect of metformin on the viability of human Hs 578T and MDA-MB-231 cells, which are TNBC cells. To this end, cells were cultured under normoglycemic conditions in DMEM supplemented with 10% FBS and 5 mM glucose. The cells were then exposed to various concentrations of metformin (1–10 mM) for 24 to 48 h, after which cell viability was assessed using the MTT assay. Metformin inhibited the growth of both Hs 578T and MDA-MB-231 cells in a dose-dependent manner. After 24 h of 5 mM metformin treatment, the proportions of live Hs 578T cells relative to control cells were 99.3%, 92.4%, 85.6%, and 76.3% (*P* < 0.001), whereas the viability values for MDA-MB-231 cells were 98.3%, 92.4%, 80.3%, and 68% (*P* < 0.001) (Fig. 1a, b). We further investigated whether a combination of metformin and cisplatin elicits a synergistic effect on cell proliferation. The combined treatment decreased cell viability compared to treatment with either metformin or cisplatin alone (Fig. 1c, d), and decreased viability significantly more than treatment with metformin alone. Next, we examined the effect of co-treating cells with cisplatin and metformin on migration and invasion by performing wound healing and invasion assays. We found that cisplatin and metformin co-treatment significantly reduced cell migration (Fig. 1e, f) and invasion (Fig. 1g, h). Taken together, these results demonstrate that metformin enhances the cisplatin-mediated antiproliferative effects in human TNBC cells.

### Metformin decreases cisplatin-induced upregulation of RAD51 expression

**Fig. 2:**
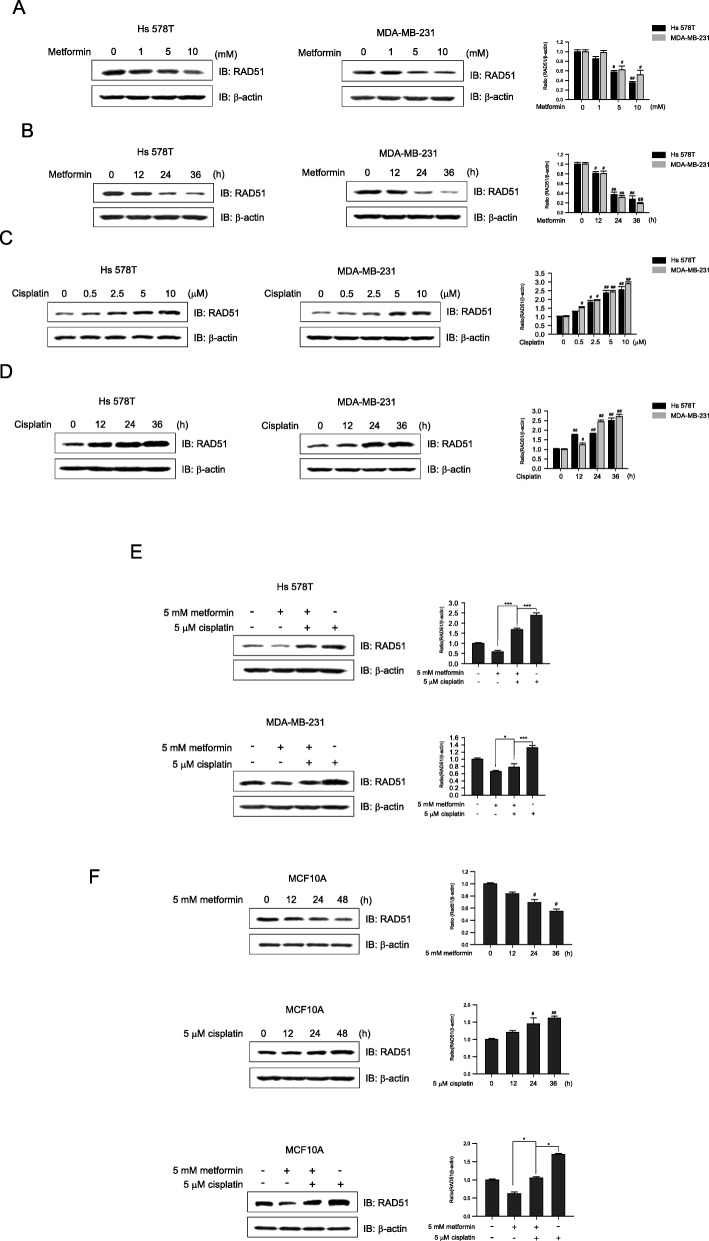
Effects of metformin, cisplatin, or a combination thereof on RAD51 protein expression. Western blotting was performed to determine the effects of metformin, cisplatin, or their combination on RAD51 protein expression. β-actin was used as a loading control. Band intensities were quantified and are presented as bar graphs. **a** Dose-dependent effect of metformin on RAD51 protein expression was determined in Hs 578T (left panel) and MDA-MB-231 (right panel) cells treated with metformin (1~10 mM) for 24 h. **b** Time course of metformin effects on RAD51 protein expression was determined in Hs 578T (left panel) and MDA-MB-231 (right panel) cells treated with 5 mM metformin for 0, 12, 24, or 36 h. **c** Dose-dependent effect of cisplatin on RAD51 protein expression in Hs 578T (left panel) and MDA-MB-231 (right panel) cells treated with cisplatin (0.5~10 μM) for 24 h. **d** Time course of the effect of cisplatin on RAD51 protein expression in Hs 578T (left panel) and MDA-MB-231 (right panel) cells treated with 5 μM cisplatin for 0, 12, 24, or 48 h. **e** Hs 578T (upper panel) and MDA-MB-231 (lower panel) cells were treated with metformin (5 mM), cisplatin (5 μM), or a combination thereof for 24 h. **f** MCF10A cells were treated with 5 mM metformin (upper panel) or 5 μM cisplatin (middle panel) for 0, 12, 24, or 48 h. In a separate experiment, MCF10A cells were treated with metformin (5 mM), cisplatin (5 μM), or a combination thereof (lower panel) for 48 h. Results represent the mean ± SEM of five independent experiments. #, ##, ### vs. no treatment; ^#^*P* < 0.05, ^##^*P* < 0.01, ^###^*P* < 0.001, *P < 0.05, **P < 0.01 by one-way ANOVA followed by Bonferroni’s post hoc test. NS: not significant

To examine the effect of metformin on RAD51 expression, Hs 578T and MDA-MB-231 cells were treated with metformin and analyzed by western blotting. The RAD51 level decreased in a dose- and time-dependent manner following treatment with metformin alone (Fig. 2a, b). In contrast, the level of RAD51 increased in a dose- and time-dependent manner following treatment with cisplatin alone (Fig. 2c, d). To determine the effect of metformin on cisplatin-mediated upregulation of RAD51, cells were treated with cisplatin in the presence or absence of metformin. Interestingly, metformin inhibited the cisplatin-mediated upregulation of RAD51 in the two cell lines (Fig. 2e). Concurrently, the effect of metformin and cisplatin co-treatment on RAD51 expression was analyzed in the MCF10A human normal breast epithelial cell line (Fig. 2f). Metformin suppressed the RAD51 protein levels in MCF10A cells, whereas the expression of RAD51 increased time-dependently with cisplatin treatment alone. Metformin also suppressed the cisplatin-mediated increase in RAD51 protein levels in MCF10A cells. Combined, our results demonstrate that metformin downregulates the cisplatin-mediated increase in RAD51 expression in both breast cancer and normal mammary epithelial cells.

### Metformin decreases the stability of the RAD51 protein

**Fig. 3:**
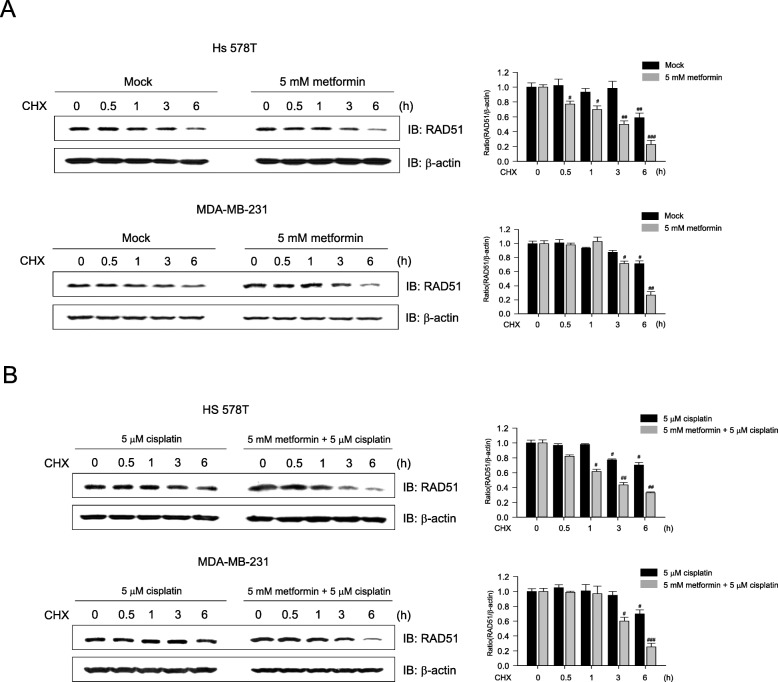
Metformin decreases RAD51 protein stability. **a** Hs 578T and MDA-MB-231 cells were pretreated with 5 mM metformin or control diluent (mock) for 24 h, followed by 5 μM cycloheximide (CHX) treatment for 0.5 to 6 h to block protein synthesis. The cells were harvested at the indicated time points after CHX treatment and immunoblotted for RAD51 or β-actin (loading control). Results represent the mean ± SEM of five independent experiments. #, ## vs. CHX treatment alone. **b** Hs 578T and MDA-MB-231 cells were pretreated with cisplatin (5 μM) or metformin (5 mM) for 24 h, followed by CHX treatment (5 μM). Results represent the mean ± SEM of five independent experiments. #, ## vs. cisplatin treatment alone; ^#^*P* < 0.05, ^##^*P* < 0.01 by one-way ANOVA followed by Bonferroni’s post hoc test

To investigate whether the metformin-mediated downregulation of RAD51 occurred at the post-translational level, cells were co-treated with metformin and CHX, an inhibitor of de novo protein synthesis, for 0.5 to 6 h. The RAD51 level declined gradually in the presence of CHX; in addition, metformin enhanced RAD51 degradation in the presence of CHX in both Hs 578T and MDA-MB-231 cells. In Hs 578T and MDA-MB-231 cells, 58.8% and 71.26% of the initial RAD51 concentrations, respectively, remained in untreated cells, whereas only 22.7% and 26.82% remained in metformin-treated cells compared to control cells. This indicates that RAD51 was less stable after metformin treatment (Fig. 3a). To test if metformin is involved in cisplatin-induced RAD51 stability, Hs 578T and MDA-MB-231 cells were co-treated with metformin and cisplatin in the presence of CHX. As shown in Fig. 3b, the combination therapy further decreased RAD51 stability in these cells. These findings suggest that metformin inhibits cisplatin-induced RAD51 expression by reducing RAD51 stability.

### Metformin induces proteasomal degradation of RAD51

**Fig. 4:**
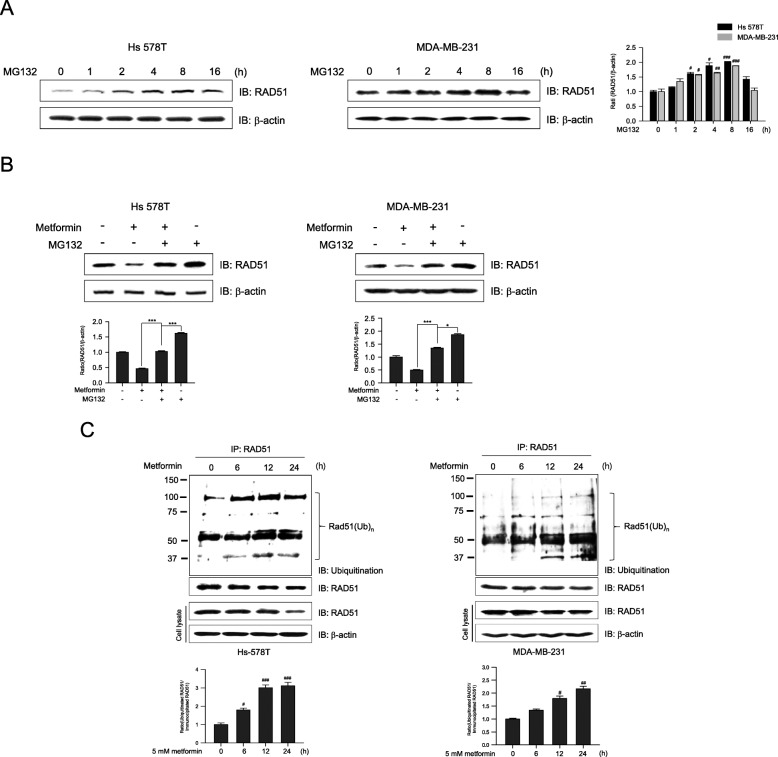
Metformin decreases RAD51 levels via ubiquitin/26S proteasome-mediated proteolysis. **a** Hs 578T and MDA-MB-231 cells were treated with 5 μM MG132, a proteasome inhibitor, for 0, 1, 2, 4, 8, or 16 h and harvested at the indicated time points for western blot analyses of RAD51 expression. Results represent mean ± SEM of five independent experiments. #, ##, ### vs. no treatment; ^#^*P* < 0.05, ^##^*P* < 0.01, ^###^*P* < 0.001 by one-way ANOVA followed by Bonferroni’s post hoc test. **b** Hs 578T (left panel) and MDA-MB-231 (right panel) cells were incubated with metformin (5 mM) for 24 h prior to MG132 (5 μM) treatment for 8 h. Cells were immunoblotted for RAD51 or β-actin (loading control). **c** Hs 578T (upper panel) and MDA-MB-231 (lower panel) cells were treated with metformin (5 mM) for 0, 6, 12, or 24 h. Protein complexes in the cell lysates were immunoprecipitated with anti-RAD51 antibodies, followed by immunoblotting with anti-ubiquitin antibodies. Whole cell lysates were immunoblotted to determine RAD51 and β-actin expression. Results represent the mean ± SEM of five independent experiments. #, ## vs. no treatment; ^#^*P* < 0.05, ^##^*P* < 0.01, **P < 0.01 by one-way ANOVA followed by Bonferroni’s post hoc test

Downregulation of RAD51 levels by metformin could be a result of increased RAD51 degradation. To investigate this possibility, Hs 578T and MDA-MB-231 cells were treated with the proteasome inhibitor MG132. The Rad51 expression was increased significantly at 2 h and maximally at 8 h after MG132 treatment in Hs 578T and MDA-MB-231 (Fig. 4a), suggesting that RAD51 degradation in Hs 578T and MDA-MB-231 cells is proteasome-dependent. We next assessed whether metformin affects the proteasomal degradation of RAD51. As shown in Fig. 4b, combined treatment with MG132 and metformin abrogated the metformin-induced RAD51 downregulation in these cells, suggesting that the ubiquitin-proteasome pathway may be involved in metformin-mediated downregulation of RAD51. In addition, we investigated whether ubiquitination of RAD51 was directly regulated by metformin in the two cell lines. After treatment with metformin for different durations, RAD51 ubiquitination increased in a time-dependent manner (Fig. 4c). These results clearly demonstrate that RAD51 proteolysis leads to reduced RAD51 levels following treatment with metformin.

### Metformin regulates the expression of RAD51 via the ERK pathway

**Fig. 5:**
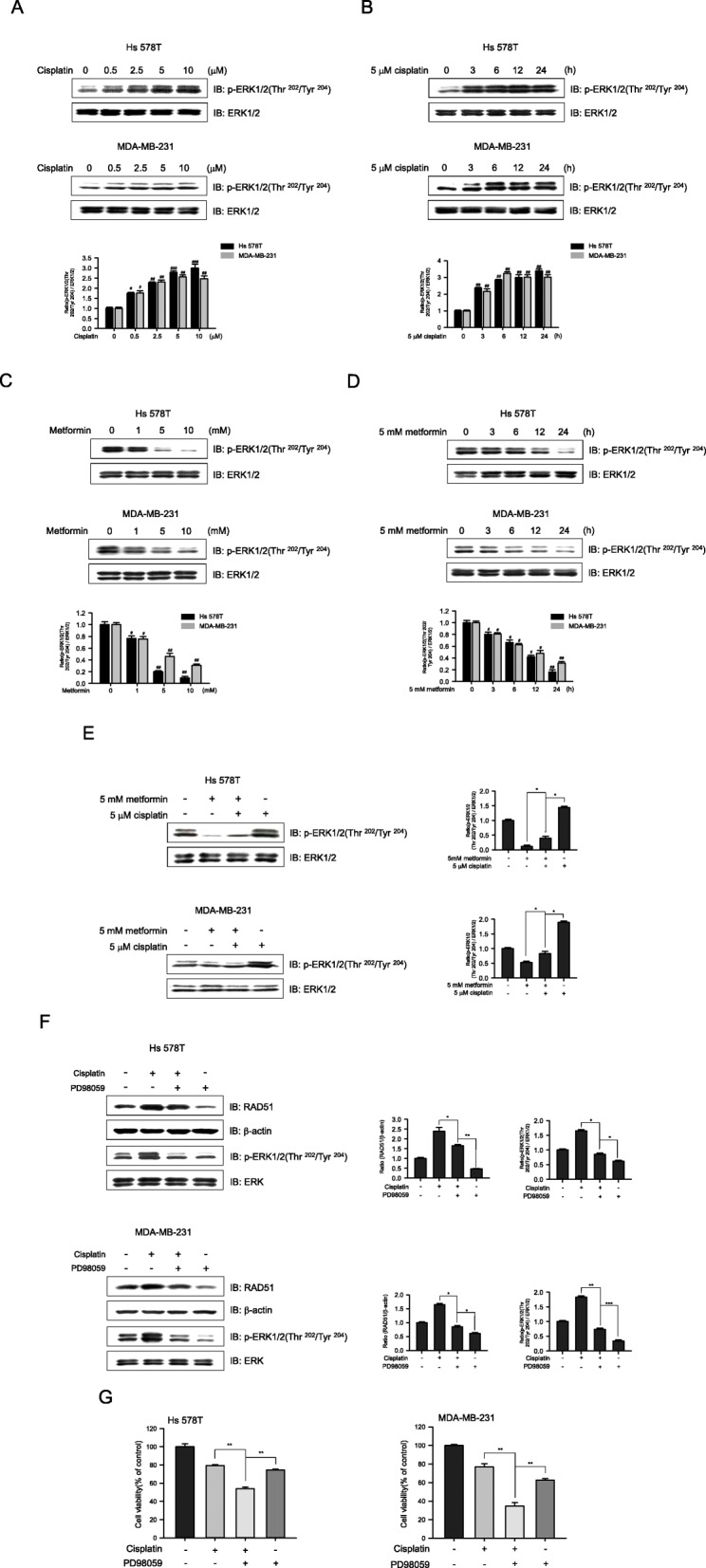
Metformin regulates RAD51 expression through the ERK pathway. **a**, **b** Hs 578T and MDA-MB-231 cells were treated with **a** 0.5~10 μM cisplatin and cultured for 6 h or **b** 5 μM cisplatin and cultured for the indicated times. Subsequently, 30-μg samples of whole cell lysates were subjected to western blotting using an antibody against phospho-ERK1/2 (Thr^202^/Tyr^204^) or total ERK1/2 (control). Bar graphs show immunoblotting band intensities. **c**, **d** Hs 578T and MDA-MB-231 cells were treated with metformin (1~10 mM) for 6 h (**c**) or with metformin (5 mM) for 0, 3, 6, 12, or 24 h (**d**). Whole cell lysates were subjected to immunoblotting for phospho-ERK1/2 (Thr^202^/Tyr^204^) and total ERK1/2. **e** Hs 578T and MDA-MB-231 cells were treated for 6 h with metformin (5 mM) and cisplatin (5 μM), either alone or in combination. Whole cell lysates were subjected to immunoblotting for phospho-ERK1/2 (Thr^202^/Tyr^204^) and total ERK1/2. **f** Hs 578T (upper panel) and MDA-MB-231 (lower panel) cells were treated with cisplatin (5 μM) for 24 h after pretreatment for 30 min with 30 μM PD98059, an ERK-specific inhibitor. Whole cell lysates were subjected to immunoblotting for phospho-ERK1/2 (Thr^202^/Tyr^204^), RAD51, total ERK1/2, or β-actin (loading control). **g** Hs 578T (left panel) and MDA-MB-231 (right panel) cells were pretreated with PD98059 (30 μM) for 30 min and then incubated with cisplatin (5 μM) for 24 h. Cell viability was determined by MTT assay. Results represent the mean ± SEM of five independent experiments. #, ## vs. no treatment; ^#^*P* < 0.05, ^##^*P* < 0.01, *P < 0.05, **P < 0.01 by one-way ANOVA followed by Bonferroni’s post hoc test

Extracellular signal-regulated kinases 1/2 (ERK1/2) have been reported to regulate RAD51 expression [33, 34]. To examine whether the cisplatin-mediated induction of RAD51 in Hs 578T and MDA-MB-231 cells was a result of ERK1/2 activation, these cells were treated with cisplatin. Treatment with cisplatin resulted in a dose- and time-dependent increase in ERK1/2 phosphorylation (Fig. 5a, b), whereas metformin decreased ERK1/2 phosphorylation in a dose- and time-dependent manner (Fig. 5c, d). To investigate the effect of metformin on cisplatin-induced phosphorylation of ERK1/2, we quantified the ERK1/2 phosphorylation levels after treatment with metformin, cisplatin, or metformin and cisplatin. Co-treatment suppressed the cisplatin-induced phosphorylation of ERK1/2 (Fig. 5e). To further elucidate the role of the ERK1/2 pathway in RAD51 expression, the two cell lines were treated with PD98059, a MEK (mitogen-activated protein kinase) inhibitor that functions upstream of ERK. Inactivating ERK1/2 blocked cisplatin-induced RAD51 expression, suggesting that cisplatin induces RAD51 expression via the MEK-ERK1/2 pathway (Fig. 5f). In addition, ERK1/2 inhibition attenuated cell viability more efficiently than treatment with cisplatin alone (Fig. 5g). Taken together, our data indicate that the ERK pathway is involved in the metformin-mediated regulation of RAD51.

### Metformin enhances cisplatin-induced DNA damage

**Fig. 6:**
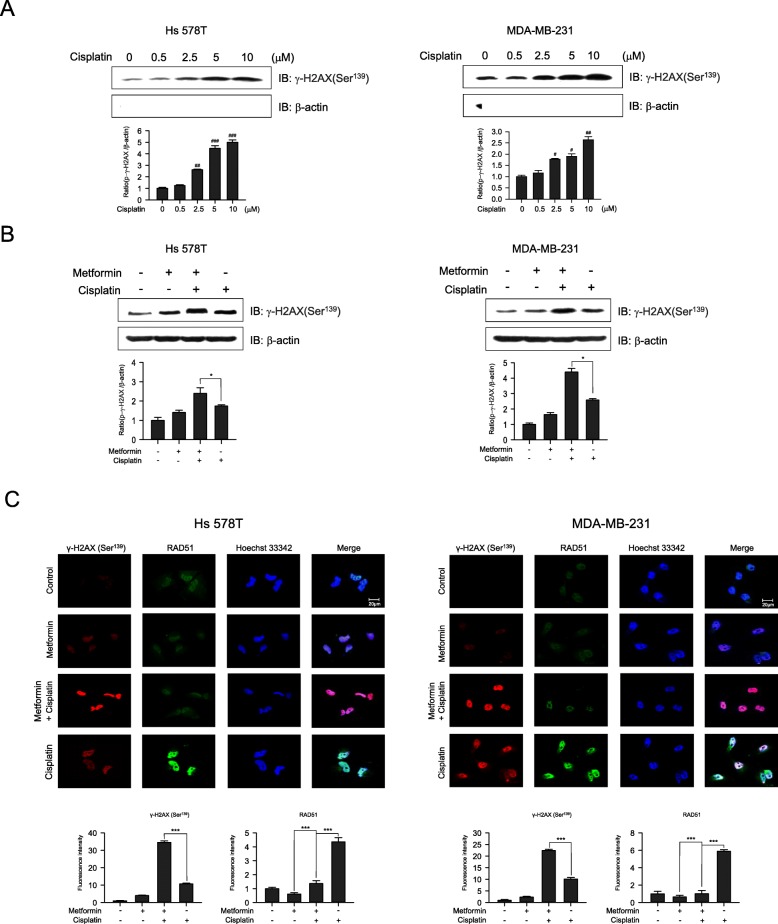
Metformin enhances cisplatin-induced DNA damage. **a**, **b** Western blotting analyses of phospho-Ser^139^ H2AX expression in Hs 578T and MDA-MB-231 cells treated with cisplatin, metformin, or their combination. **a** Cells were exposed to cisplatin (0.5~10 μM) for 6 h. **b** Cells were treated with metformin (5 mM) and cisplatin (5 μM), either alone or in combination, for 6 h. **c** Immunofluorescence staining of Hs 578T and MDA-MB-231 cells after treatment with metformin (5 mM) and cisplatin (5 μM), either alone or in combination, for 24 h. Cells were immunostained with antibodies against γ-H2AX (Ser^139^) (red) and RAD51 (green), as well as with Hoechst 33342 (blue; nucleus). Scale bars = 20 μm. Results represent the mean ± SEM of five independent experiments. #, ##, ## vs. no treatment; ^#^*P* < 0.05, ^##^*P* < 0.01, ^###^*P* < 0.001, *P < 0.05, **P < 0.01, ***P < 0.001 by one-way ANOVA followed by Bonferroni’s post hoc test

We investigated the effects of cisplatin on DNA damage as phosphorylated H2AX (γ-H2AX) is known to play a role in the retention of repair and signaling factor complexes at sites of DNA damage [35, 36]. Cells were treated with vehicle, 0.5, 2.5, 5, or 10 μM cisplatin for 6 h, and cellular extracts were analyzed for the presence of γ-H2AX. As shown in Fig. 6a, cisplatin treatment significantly increased γ-H2AX levels compared to no treatment. To explore whether metformin enhances cisplatin-induced DNA damage, the levels of γ-H2AX were measured after co-treatment with cisplatin and metformin. Cells treated with metformin and cisplatin exhibited greatly increased γ-H2AX levels compared to metformin or cisplatin treatment alone (Fig. 6b). To further investigate the synergistic effect of metformin on cisplatin-induced DNA DSBs, immunocytochemical analysis was performed with γ-H2AX and RAD51 antibodies. The γ-H2AX level increased significantly following metformin and cisplatin co-treatment, whereas that of RAD51 declined after combination treatment when compared to cisplatin treatment alone (Fig. 6c). This suggests that the reduced HR activity resulting from decreased RAD51 levels may affect DNA repair, and high levels of γ-H2AX may suggest defective DNA repair. Together, these results indicate that metformin suppresses the repair of cisplatin-mediated DNA damage in Hs 578T and MDA-MB-231 cells.

### Metformin enhances the cisplatin-mediated inhibition of migration and metastasis via RAD51

**Fig. 7:**
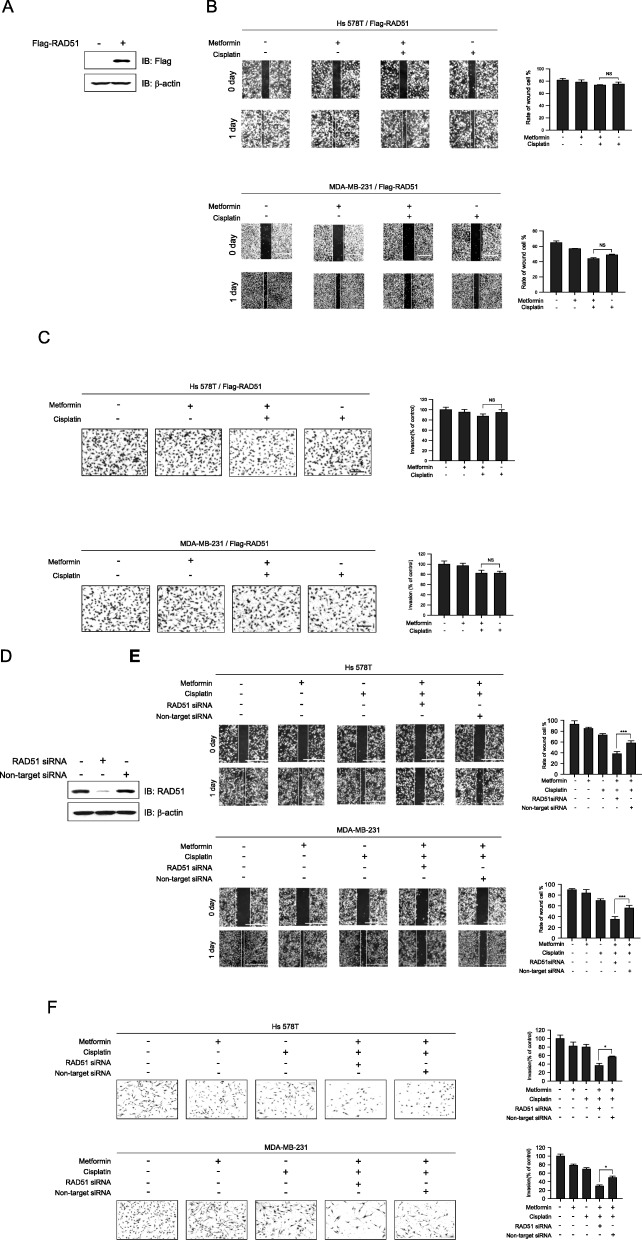
RAD51 regulates metformin- and cisplatin-mediated cell invasion. **a** MDA-MB-231 cells were transfected with a RAD51-flag expression plasmid for 48 h, followed by western blotting using antibodies against RAD51 and β-actin (control). **b** Hs 578T and MDA-MB-231 cells overexpressing RAD51 were seeded into a six-well plate for a scratch wound migration assay. Cells were treated with metformin (5 mM) and cisplatin (5 μM), either alone or in combination, for 12 h. **c** Cell invasion was measured by the Matrigel Transwell assay following treatment with metformin (5 mM), cisplatin (5 μM), or a combination of both, and quantified by staining and counting viable cells on the lower surface of the Transwell. **d** MDA-MB-231 cells were transfected with either a scrambled sequence control or RAD51-targeting siRNA for 48 h. Western blotting was performed using antibodies against RAD51 and β-actin to confirm the reduction in RAD51 levels. **e** Hs 578T (upper panel) and MDA-MB-231 (lower panel) cells were transfected with non-target or RAD51 siRNA for 24 h. Cultures of Hs 578T and MDA-MB-231 cells were wounded by scratching with a pipette tip and incubated with metformin (5 mM), cisplatin (5 μM), or a combination of both. Representative images of wound healing were obtained at the time of the scratch and after 24 h. Bar graphs (*n* = 5) are shown. **f** Hs 578T (upper panel) and MDA-MB-231 (lower panel) cells were transfected with non-target or RAD51 siRNA for 24 h. Invasiveness of RAD51 knockdown or control cells was evaluated using the Matrigel-covered Transwell invasion assay following treatment with metformin (5 mM), cisplatin (5 μM), or a combination of both. Cell invasion was quantified by staining and counting viable cells on the lower surface of the Transwell. Results represent the mean ± SEM of five independent experiments. #, ##, ## vs. no treatment; ^#^*P* < 0.05, ^##^*P* < 0.01, ^###^*P* < 0.001, *P < 0.05, **P < 0.01, **P < 0.001 by one-way ANOVA followed by Bonferroni’s post hoc test

RAD51 is required for the metastatic expansion and progression of TNBC cells [22]. Since we found that combination treatment inhibits migration and invasion to a greater extent than treatment with metformin or cisplatin alone (Fig. 1e–h), we examined if RAD51 was responsible for the synergistic effect of metformin and cisplatin co-treatment by knockdown or overexpression of RAD51. We first confirmed that RAD51-flag was overexpressed in MDA-MB-231 cells (Fig. 7a). As shown in Fig. 7 b and c, RAD51 overexpression abrogated the inhibition of migration and invasion in both MDA-MB-231 and Hs 578T cells after combination treatment. RAD51 expression decreased after transfection of RAD51 siRNA (Fig. 7d). However, cisplatin and metformin did not have a synergistic effect on migration and invasion following downregulation of RAD51 (Fig. 7e, f). These results suggest that RAD51 plays a role in the anticancer effects of combined metformin and cisplatin treatment.

### Metformin potentiates the efficacy of cisplatin in BALB/c mice injected with 4T1 cells

**Fig. 8:**
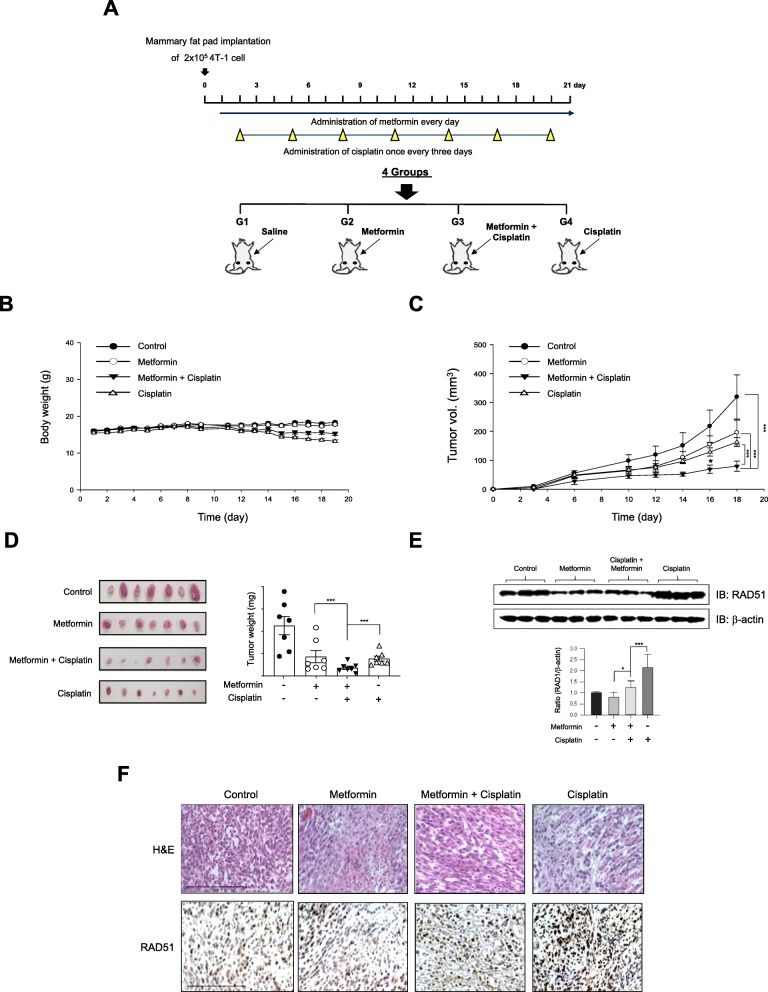
Metformin potentiates cisplatin-mediated inhibition of breast cancer growth in an orthotopic murine breast cancer model. **a** Schematic representation of the experiment. Briefly, 4T1 murine breast cancer cells were implanted in the mammary fat pad of female BALB/c mice, following which the mice were randomly allocated to four treatment groups. Treatments comprised administration of saline, metformin (IP, 150 mg/kg, daily), cisplatin (IP, 3 mg/ kg, q3d), or a combination of both for 3 weeks. **b** The body weight of the mice was measured daily. Data represent the mean ± SEM (*n* = 10/group). No significant difference was observed (*P* > 0.05). **c** Tumor growth was monitored by measuring tumor size with calipers every other day. Data represent the mean ± SEM (*n* = 10/group). ***P < 0.001 by one-way ANOVA followed by Bonferroni’s post hoc test. **d** Net tumor weights and representative tumor images upon necropsy are shown. Statistical analyses were performed using GraphPad Prism 7. All data were tested for normality by the Shapiro-Wilk test. Student’s *t* test (for normally distributed samples) and the Mann-Whitney *U* test (for nonparametric analyses) were performed to compare groups. All statistical analyses were two-tailed. Linear regression analysis was performed to test whether slopes and intercepts in tumor growth curves were significantly different. **e** Tumor lysates were analyzed for RAD51 expression by western blot. The bar graph represents quantification of band intensities (*n* = 3) *P < 0.05, ***P < 0.001 based on one-way ANOVA followed by Bonferroni’s post hoc test. **f** Immunohistochemical staining of the tumors confirms RAD51 expression (× 200 magnification)

To further validate our in vitro findings, we investigated the effects of combination treatment in a 4T1 murine breast cancer model using BALB/c mice. The experimental procedure is described in the “Methods” section and is shown in Fig. 8a. No significant changes in body weight were observed among the groups (Fig. 8b). The average tumor volume in the control group increased gradually, reaching 98 ± 312 mm^3^ on day 21 after implantation, whereas that in the combination group was significantly inhibited (46 ± 79.5 mm^3^, *P* < 0.05; Fig. 8c). The average tumor weight in each group was determined after all the mice had been euthanized. Average orthotopic tumor weights were 320, 285, 98, and 160 mg for the control, metformin, combination cisplatin and metformin, and cisplatin groups, respectively (Fig. 8d). The average tumor weight was lowest in the combination treatment group. The western blot results demonstrated that RAD51 levels were significantly decreased in the combination treatment group compared to the other groups (Fig. 8e). The RAD51 immunostaining results in tumor tissue correlated with those of the western blot analysis (Fig. 8f), indicating that RAD51 is a key molecule in chemosensitization to cisplatin.

## Discussion

Cisplatin resistance limits therapeutic options in patients diagnosed with TNBC. The main objectives of our study were to determine if metformin sensitized human TNBC cells to cisplatin and, if so, to identify the molecular signaling pathways involved. The principal findings of our study were that metformin acted as a cisplatin sensitizer in TNBC chemotherapy and that RAD51 played a critical role in the synergistic effect of metformin on cisplatin. Consequently, RAD51 represents a potential therapeutic target in TNBC patients.

Although single-agent therapy has yielded positive results in cell lines and preclinical models, it failed to show promising results in managing aggressive TNBC in clinical trials, likely due to therapy heterogeneity and potential for acquired drug resistance [37]. Several studies have shown that combining metformin with cisplatin is effective in treating various cancers, including ovarian carcinoma [29], human nasopharyngeal cell carcinoma [30], lung carcinoma [31], and oral squamous cell carcinoma [32]. In addition, metformin reduces cisplatin-induced side effects like cognitive impairment, brain damage [38], and peripheral neuropathy [39] in mice. This is the first study exploring the chemosensitizing effect of metformin on cisplatin against TNBC cells through the regulation of DNA damage repair.

In this study, we found that metformin sensitized MDA-MB-231 and Hs 578T TNBC cells to cisplatin based on cell viability (Fig. 1c, d). Metformin also enhanced cisplatin-mediated inhibition of migration and invasion (Fig. 1e–h). Our results indicate that the anticancer effects of metformin under reduced glucose were more pronounced in MDA-MB-231 than HS-578T cells. Most in vitro studies have shown the efficacy of metformin as an anticancer agent using very high concentrations (> 5 mM), which may be due to the high glucose concentrations used in the culture of most cancer cell lines. The presence of glucose at high concentrations reduced the antineoplastic efficacy of metformin, indicating that investigations on the anticancer effects of metformin should be performed under physiologically relevant glucose concentrations. Metformin also exhibited significant biological activity in a 4T1 mouse breast cancer model in vivo. In mice with normal levels of glucose and insulin, combined metformin and cisplatin treatment decreased the tumor volume to a significantly greater extent than cisplatin treatment alone (Fig. 8c, d), suggesting that metformin has potential as a therapeutic agent against TNBC in combination with cisplatin.

However, for successful clinical application, a few limitations should be considered. First, it is still unknown whether the anticancer effects of metformin are replicated in clinical models. Therefore, studies are necessary to determine the most appropriate dose and establish the safety of metformin in patients with TNBC. Second, although metformin is used as the first-line treatment for type 2 diabetes, the appropriate range for its therapeutic concentration is still confounding. According to previous studies, a range of approximately 5 mM metformin was effective in breast cancer cell lines [40, 41]. Moreover, metformin accumulated and reached tissue concentrations substantially higher than those found in the plasma [42], implying that the therapeutic metformin plasma concentration might be lower than that for tissue. Therefore, the metformin concentration (5 mM) used in the present study seems appropriate and is considered relevant for use in vitro studies.

Elevated expression of RAD51 is associated with tumor aggressiveness and is known to confer treatment resistance in a variety of tumors, including ovarian cancer [43], breast cancer [44], lung tumors [45], pancreatic adenocarcinomas [46], and malignant gliomas [47]. Furthermore, downregulation of RAD51 protein levels by antisense oligonucleotides, RNA interference [48], aptamers [49], or small-molecule inhibitors can be used to sensitize tumors to chemotherapy or radiation. In this study, we found that RAD51 expression increased in a dose- and time-dependent manner following cisplatin treatment, whereas it decreased in a dose- and time-dependent manner with metformin treatment (Fig. 2a–d). Interestingly, metformin inhibited cisplatin-mediated RAD51 upregulation (Fig. 2e), indicating that the metformin-mediated downregulation of RAD51 may inhibit resistance to cisplatin in TNBC cells. We further investigated the effect of metformin on the normal breast epithelial cells, MCF10A. Metformin decreased the expression of RAD51 and inhibited the cisplatin-mediated RAD51 expression in MCF10A (Fig. 2f). Previous reports showed that extracellular vesicles (EVs) from triple-negative breast cancer cells promoted proliferation and drug resistance in MCF-10A [50, 51], implying that TNBC-mediated EVs (TNBC-EVs) may induce tumorigenic potentiality in normal cells. Combined with the result of Fig. 2f, metformin may reduce cisplatin resistance induced by TNBC-EVs in normal tissues via RAD51. In addition, it was reported that metformin selectively targeted cancer stem cells and also induced apoptosis in human breast carcinoma cell line MCF-7 with minimal toxicity to MCF10A [52, 53]. Furthermore, metformin prevented normal cell apoptosis against cisplatin-induced ototoxicity and nephrotoxicity in auditory cell and tubular cell [54]. Together, these findings indicate that metformin may be a potentially adjuvant therapy drug to combine with cisplatin. In the future, in-depth studies are necessary to determine appropriate modes of combination therapy of metformin and cisplatin.

Moreover, we confirmed the effect of RAD51 on the metformin-induced inhibition of migration and invasion after knock down or overexpression of RAD51 using RAD51 siRNA and RAD51-flag. As expected, RAD51 overexpression blocked metformin-mediated inhibition of migration and invasion while its downregulation enhanced the effect of metformin (Fig. 7e, f). This suggests that RAD51 is a potential therapeutic target for TNBC treatment. In support of our findings, studies have shown that RAD51 overexpression contributes to chemoresistance in human soft tissue sarcoma cells [55] and rescues radiation sensitivity in BRCA2-defective cancer cells [56].

Double-strand breaks represent one of the most important types of cisplatin-induced DNA damage. In response to DSBs, histone H2AX is rapidly activated and phosphorylated, generating γ-H2AX. In this study, metformin enhanced the cisplatin-mediated phosphorylation of γ-H2AX (Fig. 6b, c), suggesting that metformin prolongs the process of cisplatin-induced DSB repair and regulates the γ-H2AX-RAD51 axis to overcome resistance to cisplatin.

Reduced food intake and weight loss are serious health concerns in patients undergoing cisplatin therapy [57]. In this study, cisplatin treatment resulted in progressive weight loss. Interestingly, however, metformin and cisplatin combination treatment attenuated the cisplatin-mediated weight loss (Fig. 8b). Our data demonstrated that metformin attenuates cisplatin-induced side effects and potentiates cisplatin-mediated anticancer effects.

## Conclusions

In conclusion, metformin effectively enhanced the anticancer effects of cisplatin. This effect of metformin is likely mediated through the downregulation of RAD51, a key player in HR repair, leading to defective DSB repair. Our in vitro results, together with our orthotopic 4T1 mouse model results, demonstrate that metformin may potentially act as a cisplatin sensitizer in TNBC chemotherapy.

## Data Availability

All data generated for this study are included in the article.

## Abbreviations

ANOVA: Analysis of variance
CHX: Cycloheximide
ER: Estrogen steroid receptor
ERK: Extracellular signal-regulated kinase
HER: Human epidermal growth factor receptor 2
HR: Homologous recombination
MEK: Mitogen-activated protein kinase
PR: Progesterone steroid receptor
TNBC: Triple-negative breast cancer
γ-H2AX: Gamma-H2A histone family member
